# Evaluation of a tailored implementation strategy to improve the management of patients with chronic obstructive pulmonary disease in primary care: a study protocol of a cluster randomized trial

**DOI:** 10.1186/1745-6215-15-109

**Published:** 2014-04-04

**Authors:** Maciek Godycki-Cwirko, Izabela Zakowska, Katarzyna Kosiek, Michel Wensing, Jaroslaw Krawczyk, Anna Kowalczyk

**Affiliations:** 1 Centre for Family and Community Medicine, Medical University of Lodz, 20 Kopcinskiego Street, Lodz 90-153, Poland; 2 Radboud University Medical Centre, Scientific Institute for Quality of Healthcare, P.O. Box 9101, 114, Nijmegen 6500, HB, The Netherlands

**Keywords:** Chronic obstructive pulmonary disease, Implementation science, Primary health care

## Abstract

**Background:**

Chronic obstructive pulmonary disease (COPD) remains a major health problem, strongly related to smoking. Despite the publication of practice guidelines on prevention and treatment, not all patients with the disease receive the recommended healthcare, particularly with regard to smoking cessation advice where applicable. We have developed a tailored implementation strategy for enhancing general practitioners’ adherence to the disease management guidelines. The primary aim of the study is to evaluate the effects of this tailored implementation intervention on general practitioners’ adherence to guidelines.

**Methods/Design:**

A pragmatic two-arm cluster randomized trial has been planned to compare care following the implementation of tailored interventions of four recommendations in COPD patients against usual care. The study will involve 18 general practices (9 in the intervention group and 9 in the control group) in Poland, each with at least 80 identified (at the baseline) patients with diagnosed COPD. The nine control practices will provide usual care without any interventions. Tailored interventions to implement four recommendations will be delivered in the remaining nine practices. At follow-up after nine months, data will be collected for all 18 general practices. The primary outcome measure is physicians’ adherence to all four recommendations: brief anti-smoking advice, dyspnea assessment, care checklist utilization and demonstration to patients of correct inhaler use. This measurement will be based on data extracted from identified patients’ records. Additionally, we will survey and interview patients with chronic obstructive pulmonary disease about the process of care.

**Discussion:**

The results of this trial will be directly applicable to primary care in Poland and add to the growing body of evidence on interventions to improve chronic illness care.

**Trial registration:**

This trial has been registered with Clinical Trials Protocol Registration System. Trial number: NCT01893476.

## Background

Chronic obstructive pulmonary disease (COPD) remains a major health problem. Worldwide it has been ranked as the sixth leading cause of death for both genders [1]. In 2020 COPD is projected to rank fifth worldwide in burden of disease. It is also projected to be the fourth leading cause of death worldwide by 2030 due to an increase in smoking rates and demographic changes in many countries [2]. A national survey conducted in Poland in 2007 indicated that 34% of men smoked daily, 2% were occasional smokers, 19% were former smokers and 45% had never smoked. In women these percentages were 23, 3, 10 and 64% respectively [3].

Epidemiological studies of COPD on a representative sample have not been performed in Poland, but estimates from smaller studies suggested a relatively high prevalence. Studies in selected geographical areas found that signs and symptoms of COPD were seen in about 10% of patients over 40-years old [4]. Studies in large cities in Poland showed a 9.8% prevalence of COPD in populations between 41 and 72 years old [5]. These data are similar to other European data, describing prevalence rates of 4 to 11% in adults in Europe [6]. The total number of people suffering from COPD in Poland is estimated to be about 2 million (on a population of 38 million inhabitants). This places COPD as the third most frequent chronic illness, and it is the fourth most common cause of death in Poland [7].

In Poland, most COPD patients are treated in primary care, which is the entry point to the public health care system. The system is based on compulsory health insurance, managed by the National Health Fund (NHF), which purchases health services from physicians and health care enterprises. Patients register with a particular primary care practice, signing on to an individual general practitioner’s list of patients, and can be referred for specialist out-patient consultation or to a hospital, if needed. Ambulatory care (primary and out-patient specialist services) is provided by therapeutic entities (clinics or dispensaries) and by medical practices. Rehabilitation and long-term care are provided within both the health care sector and the social care sector, but coordination between the two is poor. Furthermore, limited financial resources available to the NHF and shortages of medical personnel have negative effects on access to health care services [8].

There is no national consensus on the care paths for COPD, and various guidelines on COPD are used, some of which national and some international [9,10]. However, the guidelines in use share most of recommendations that we have identified and prioritized for the purpose of this trial. There is very little evidence on the degree of adherence to the guidelines for the management of COPD used by Polish physicians. One study, which evaluated the NHF funded COPD prevention program aiming to reduce the incidence and disability related to COPD by complex educational, diagnostic and therapeutic interventions in people at risk, showed low rates of participation of primary health care providers despite additional funding. An interesting finding was that the extent of program completion was strongest for qualified general practitioners compared to physicians without specialty training or non-generalists: internal diseases specialists and pediatricians working at primary care setting [11].

It is unclear whether studies on the implementation of evidence-based recommendations for primary care in COPD patients from countries with well-developed primary care systems, like the United Kingdom and the Netherlands [12,13] can be translated to Poland. In searching the Polish medical literature, we did not find published research on the implementation of COPD guidelines or recommendations in Poland. The authors identified three papers with results partially related to the subject of this study [14-16]. These found that 30% of patients with the diagnosis do not fulfill criteria for COPD, in 15% of cases spirometry had not been performed, and more than 70% of patients received inhaled steroids. Such findings suggest that adherence to diagnostic and management recommendations are inadequate.

Preliminary qualitative evidence from the authors’ earlier work showed that not one single guideline for COPD was used by all GPs or medical specialists. However, responders pointed out the individual recommendations most commonly used and the barriers to their implementation. The identified barriers for adhering to specific recommendations were: (1) lack of knowledge on smoking cessation brief intervention, (2) dyspnea evaluation tool was unavailable, (3) lack of a care plan, and (4) lack of demonstration-inhaling devices. Other research has indicated that the treatment parameters from the (COPD) guidelines are not always measured [17-19]. The consequence is that not all patients receive recommended advice and treatment from their physicians.

This study is part of the Tailored Implementation for Chronic Diseases (TICD) international collaborative research project, which is being undertaken in Germany, Norway, Poland, the Netherlands and the United Kingdom. The aim is to develop and test methods of tailoring knowledge-implementation interventions to determinants of practice in chronic illness care [20]. In Poland, the focus is on the implementation of COPD guidelines in primary care. The research group in Poland selected four key recommendations applicable to primary care (Table 1) from the four COPD guidelines most relevant to primary care and used in Poland.

**Table 1:** Key recommendations for COPD management

A	Brief smoking cessation counseling is effective and every tobacco user should be offered stop-smoking advice at every contact with health care providers.
B	The prognosis of COPD should be estimated using the mMRC dyspnea scale.
C	The patient should be given the basic information about his/her disease, its treatment and the expected effects of applied drugs, in order to enhance that the patient takes an active, participating role in the long-term treatment.
D	The patient should be trained in the correct use of all devices which he/she will use for inhalation.

### Objective

The objective of this study is to examine the effectiveness of a tailored implementation strategy for enhancing physicians’ adherence to four recommendations for the management of COPD patients in primary care. The process of tailoring implementation interventions and the impact on the effectiveness of the strategy will also be studied.

### Research questions

The questions we will be attempting to address in this study are as follows: what is the effectiveness of a tailored implementation strategy compared to usual care in enhancing primary care physicians’ adherence to guidelines?, and how do the determinants of practice targeted by the tailored implementation strategy change over time, and how does their variability relate to the effectiveness of implementation strategies?

## Methods/design

### Study design

This study is a two-arm pragmatic, cluster randomized trial (CRCT) [21,22], which compares a tailored intervention program directed at general practitioners with usual primary care. It is localized in Lodz, the third largest city in Poland, with a population of about 750.000 inhabitants with broadly average mortality and morbidity, although specific health data are lacking. It aims to include general practices with adult patients with COPD under their care. The general practices will be randomized into two equally sized groups

### Sample size calculation

The sample size calculation indicated that, for a two-arm cluster controlled randomized trial, a minimum number of 16 primary care practices with minimum of 30 COPD patients per practice is required. A total of 16 clusters and at least 480 subjects is required at baseline and follow-up (8 clusters in each study arm) to detect a difference, or change in adherence, of between 40% and 60% with selected COPD recommendations, between the intervention group (facilitating adherence to guidelines) and the control group without intervention (usual care), with 80% power, a two-tailed alpha of 0.05, and intra-cluster correlation of 0.05.

A standard sample size formula was used to calculate the initial unadjusted sample size requirements, followed by appropriate adjustment for clustering by general practice according to Campbell *et al.*[23], with expected small clustering effect (ICC = 0.05). Previous reported ICCs vary from 0.03 for smoking advice [23] to 0.05 for cluster randomized trials in primary care (data from Trial of Older People in the Community) [24,25]. It was decided to increase the sample by 10% to account for contingencies such as non-response or recording error, giving a total of 18 practices.

With regard to the number of patients with COPD per practice, an assumed response rate of 58% [26,27] and a dropout of 35% [28], which led to a total of 80 COPD patients needed per general practice at baseline. Patients will be selected via the medical records using ICD code J44, and medical records will be labelled at the baseline of the study.

### Setting and participants

#### General practices

**Figure 1:**
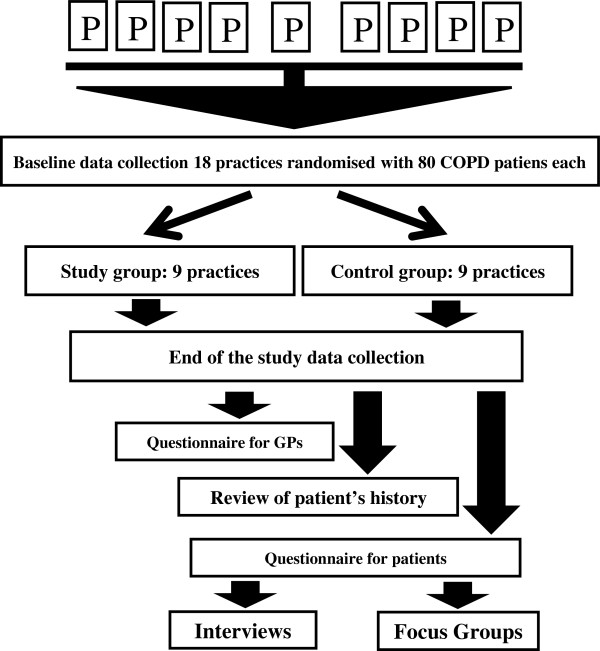
Flowchart for CRCT.

Eighteen general practices selected with random sampling within the Lodz region of Poland, with 80 or more registered COPD patients each (identified via J44 ICD-10), will be invited to participate in the study. The baseline number of COPD patients is determined by assumed response rate and dropout described above, to assure at least 30 at follow-up. Some of the practices were previously involved in other studies and have an established agreement with the Medical University of Lodz in the field of research and vocational training. The main study has been planned to take place between December 2013 and September 2014. After having given informed consent, primary care practices will be randomly allocated to one of two groups. The medical records of patients with COPD will be labelled with blue stickers at the baseline of the study (Figure 1).

#### General practitioners

All GPs working in targeted practices are required to participate in the study.

### Eligibility criteria

#### Patients

Eligible patients will be approached at follow-up. Only patients who were treated in the same practice at baseline (with the labelled medical records) will be considered for inclusion so that the study is prospective. Exclusion criteria are: (1) terminal illness and (2) cognitive impairments. At follow up 80 patients, randomly selected (with personal data unknown to researchers) will receive an informed consent form and an invitational letter via their GPs, and will be asked to fill in a questionnaire and/or participate in an audio taped interview or focus group. This letter will provide comprehensive information about the study. Contact details of the researchers will be provided so that patients can ask questions. Patients’ information gathered from questionnaires or from interviews will be anonymized.

### Randomization

Randomization will be performed by a statistician not involved in the trial, through a computer. The general practices will be allocated randomly to two equally sized groups: an intervention group and a control group. At the end of the study a group of patients will be randomly selected in order to invite them to fill out the end-of-study questionnaire. Participants in this trial will not be blinded. Descriptive statistics and logistic regression will be used for analyzing the data. The level of statistical significance will be *p* < 0.05.

### Blinding

COPD patients within each participating intervention and control practice will be identified at the baseline. Because of the nature of the intervention, it is not possible to blind GP participants (in practices).

Outcome assessment will not be blinded as the research assistants will be aware of practice group allocation, and the data analysis will be performed by researchers and a statistician blinded to the study group.

### Implementation program

The implementation program is based on multiphase research performed in the TICD project. In the first phase has identified barriers and enablers for improving COPD care, focusing on the four recommendations selected. One hundred and sixty determinants were listed, grouped (according to the TICD checklist domains [29]), and judged by our research team on importance and changeability using Likert scales. The result was 24 determinants of practice. The second phase of the project, identified determinants were prioritized during focus group discussions and matched to implementation strategies. The final decisions on the interventions matched to the determinants for four recommendations are presented in Table 2. This procedure led to the following implementation program.

**Table 2:** Identified determinants and interventions for selected recommendations

	**Recommendation**	**Determinant**	**Proposed intervention(s)**
A	A brief clinician’s counseling to quit smoking	Additional training for GPs	Labeling medical records for COPD patients and training GPs in brief intervention
B	The prognosis of COPD should be estimated using mMRC dyspnea scale	Prepared form	Additional form (in paper or in computer medical records) with mMRC scale
C	The patient should be given the basic information about his/her disease	Checklist in medical history	Providing physicians with a checklist in patients’ records to facilitate what should be done during consultation
D	The patient has to be trained in the correct use of all devices	Inhaling devices for training	To provide GPs’ clinics with training inhaler devices to show patient’s how to use them

#### Smoker identification and brief intervention

First, participating physicians will receive training in brief smoking status identification and anti-smoking counselling, and will be asked to record information about the actions they perform in patients’ medical records. This intervention will address the recommendation of Global Initiative for Chronic Obstructive Lung Disease (GOLD) based on Wilson et al. [30].

#### Dyspnea evaluation

Second, an additional form containing the modified Medical Research Council Dyspnea Scale (mMRC) will be inserted into patients’ medical records on paper. It is a validated patient symptom questionnaire for a subjective assessment of COPD symptoms. This intervention will address the recommendation of National Institute for Health and Care Excellence based on Fletcher et al. [31].

GPs will be asked to determine the patient’s status according to the scale and put this information into the patient’s medical records.

#### COPD check list

Third, a checklist for practitioners will be provided with information about what should be done while consulting on a patient with COPD. This intervention will address the recommendation of the European Respiratory Society [32]. It will cover points such as that a patient should be given basic information about COPD, its treatment, and the expected effects of applied drugs, making the patient an active, aware participant in their long-term treatment. GPs will be asked to provide patients with the information and tick a box if it is done.

#### Demonstration inhaler devices

Finally, practices will be provided with training inhaler device sets for health care staff and train GPs on how to instruct patients to use devices properly. This intervention will address the recommendation of the Polish Society of Lung Diseases [33].

GPs will be asked to teach patients in the correct use of each device and record information about the fact in patient’s medical records.

### Control group

In this arm, GPs will provide their usual care for COPD patients. The practices in the control group will receive feedback after the end of the study about their own performance in comparison with the performance of other practices in the study in relation to the guideline recommendations. The GPs will receive no intervention during the study.

### Outcomes/measures

#### Primary outcome

The primary outcome will be the GPs’ adherence to the recommendations, which is dichotomized as follows. A positive score is given if all recommendations are followed, while following less than four recommendations is given a negative score. Data listed in Table 3 will be obtained from the patients’ medical records (independent of the checklist that is provided as part of the intervention) and during interviews. All identified COPD patients who gave informed consent in each practice will be included, and full data protection procedures will be followed.

**Table 3:** Information recorded in patients’ records to determine adherence of a GP the recommendations

1.	Brief anti-smoking counselling performed
2.	Dyspnea assessed
3.	Management process of COPD assessed
4.	Training patient on correct use of inhaling device performed.

#### Secondary outcomes

The secondary outcome will be patient reported health status. We will review COPD patients’ medical records to measure health outcomes such as a change in smoking status, the quantity of COPD medications prescribed, dyspnea perception and number of exacerbations in the past and over the study period.

#### Process evaluation

All aspects of the coordinated process evaluation will be implemented in this study, following the international study protocol for the TICD project, in order to identify determinants of change in chronic illness care, to examine the validity of the tailoring methods that were applied, and to analyze the association of implementation activities with the effectiveness of the program [34]. The process evaluation will comprise three main components: a structured survey with health professionals in the trials, semi-structured interviews with a purposeful sample of this study population, and standardized documentation of organizational practice characteristics. The evaluation will be guided by ‘logic models’ of the implementation programs: frameworks that specify the linkages between the strategies used, the determinants addressed by tailoring, and the anticipated outcomes.

A written survey will be undertaken involving participating health professionals. The questionnaire will list the determinants of practice, which were identified and prioritized in an earlier phase of the TICD project, respondents being asked to assess whether the program successfully targeted them. A free text field will be used to identify possible other determinants. The survey will also contain questions on the actual implementation activities that took place, to record the extent to which the target group used the offered interventions, and any adaptions made in the delivery phase of the implementation program. The core components of the implementation program will be specified. For each of the core components, content, duration, frequency and coverage will be recorded in a structured way. The content, duration, frequency and coverage aspects of intervention fidelity will also be covered. A free text field will be added to identify strategies which have been missed in the tailoring process, allowing the evaluation of the methods used for tailoring.

The face-to-face or telephone interviews will be performed with a purposive sample of health professionals. Interim analysis after five to ten interviews will be performed to adapt the interview format and purposeful sampling scheme as required.

### Data collection

At the end of the study data will be collected by review of medical records of COPD patients identified at the baseline who visited their GPs during the last nine months. Extracted information will cover the following: brief anti-smoking advice performed, information about dyspnea, COPD checklist utilization and training on using inhalers.

Patients with COPD who have given signed informed consent will be asked to fill in a questionnaire and participate in an interview containing questions about the process of care, information received from GPs and patients’ health status perception.

### Statistical methods

The primary analysis will be on an intention to treat basis. Missing values will be inputted with a multiple imputation method. Data will be coded, cleaned and locked before any analyses are made. Quantitative data will be analyzed in aggregate, using the Statistical Package for the Social Sciences (SPSS, (version 17, IBM Corp.) and Statistica (v10, StatSoft Inc.). Study groups will be compared with respect to the determinants of COPD and its improvement. Only members of the research team who need access to the database to fulfill their roles within the study will be granted access to the database. Demographic characteristics of the practices and the participants within the practices will be described using percentages, measures of central tendency (means or medians) and measures of variation (standard deviations or ranges). The analysis will be based on the patients nested within practices two-level model. The primary endpoint is the GPs adherence to four COPD guidelines after nine months, in each practice. Secondary endpoints are the change in smoking status and health care utilization during the nine months amongst included patients. Data will be compared between arms using logistic regression (with logit link function and binomial distribution) with patient at level one and practice at level two. The ICC and odds ratios and 95% confidence intervals will be estimated using two-level random intercepts logistic regression models. The significance level will be set at α = 0.05 for all analyses.

### Ethical approval

This study has been approved by the Bioethical Committee of Medical University of Lodz, Poland (reference RNN/491/13/KB of 18 June 2013).

## Discussion

COPD care improvement in primary care has not been studied extensively. Schermer *et al.* observed a small and late effect of e-learning and repeated feedback on the quality of spirometry as performed by family practice nurses [35]. Kennedy *et al.* studied at practice-level training in a whole systems approach to self-management support for patients with chronic conditions and found no statistically significant differences between patients attending trained practices and those attending control practices, although this study looked at three conditions simultaneously (diabetes, COPD and irritable bowel syndrome) [36]. While these trials focused on specific aspects of COPD care (spirometry and self-management education, respectively), the intervention program in this study intends to improve COPD care more broadly.

Meulepas *et al.* studied the effect of a primary care model that included smoking cessation, inhaler technique training and dyspnea scoring according to the MRC scale [12]. V an den Bemt *et al.* also studied guideline based COPD-management in primary care [13]. However their interventions were at least partly directed at patients, while this study targets general practitioners. This study is probably the first randomized trial of a tailored implementation program for improving primary care for patients with chronic illness in an East European country. The study has some limitations, such as possible selection bias since practices with less than 80 COPD patients were excluded as well as those that have some GPs teachers. Nevertheless, it is the authors’ belief that the results will be directly relevant and applicable to primary care in Poland. If the implementation program is effective, then wide-scale application would be warranted.

## Trial status

Registration has been completed, general practices have been recruited and randomized, and interventions delivered.

## Abbreviations

ATS: American Thoracic Society; COPD: chronic obstructive pulmonary disease; CRCT: Cluster Randomized Controlled Trial; ERS: European Respiratory Society; EU: European Union; GOLD: Global Initiative for Chronic Obstructive Lung Disease; GP: General Practitioner; ICC: Intracluster Correlation Coefficient; ICD: International Classification of diseases; mMRC: Modified Medical Research Council; NHF: National Health Fund; NICE: National Institute on Health and Clinical Excellence; P: practice; PSLD: Polish Society on Lung Diseases; SPSS: Statistical Package for the Social Sciences; TICD: Tailored Implementation for Chronic Diseases.

## Competing interests

The authors declare that they have no competing interests.

## Authors’ contributions

MG-C and JK developed the idea and wrote the draft version of this protocol, which was commented by AK, IZ and KK. IZ did the sample size calculation. M-C corrected and rewrote consecutive versions. All authors critically assessed and approved this study protocol. Project partners discussed with and reviewed the protocol. MW revised and commented on draft versions and reviewed the final version. All authors have read and approved the manuscript for publication.

## Authors’ information

TICD is a collaborative project between five European countries (The Netherlands, UK, Germany, Poland and Norway). Michel Wensing from Radboud University, the Netherlands is the principal investigator in the TICD project. This TICD WP4 COPD trial in Poland will be coordinated by Maciek Godycki-Cwirko at the Medical University of Lodz. The researchers in the Polish team have experience with cluster randomized trials.
